# Teenagers’ mental health problems predict probable mental diagnosis 3 years later among girls, but what about the boys?

**DOI:** 10.1186/s13034-022-00473-y

**Published:** 2022-06-09

**Authors:** Kristina Carlén, Sakari Suominen, Lilly Augustine, Maiju M. Saarinen, Minna Aromaa, Päivi Rautava, André Sourander, Matti Sillanpää

**Affiliations:** 1 School of Health Sciences, University of Skövde, Box 408, 54128 Skövde, Sweden; 2 The Research School of Health and Welfare, Jönköping University, Jönköping, Sweden; 3 Department of Public Health, University of Turku and Turku University Hospital, Turku, Finland; 4 CHILD, School of Learning and Communication, Jönköping University, Jönköping, Sweden; 5 Departments of Child Neurology and General Practice, University of Turku and Turku University Hospital, Turku, Finland; 6 City of Turku Welfare Division, Turku, Finland; 7 Clinical Research Centre, Turku University Hospital, Turku, Finland; 8 Department of Child Psychiatry, University of Turku, Turku, Finland; 9 Department of Child Psychiatry, Turku University Hospital, Turku, Finland

**Keywords:** DAWBA, Internalized problems, Externalized problems, Self-report, YSR

## Abstract

**Background:**

The prevalence of mental disorders is increasing, and there seems to be a gender difference in prevalence, with girls reporting more mental health problems than boys, especially regarding internalizing problems. Most mental disorders debut early but often remain untreated into adulthood. Early detection of mental disorders is essential for successful treatment, which is not always happening. The study aimed to estimate to what extent teenagers’ self-reports predict probable mental diagnosis as they enter adulthood, particularly regarding gender differences.

**Methods:**

Self-reported mental health problems, Youth Self-Report (YSR) at 15 years (range 3–110, n = 504) from the ongoing Finnish family competence study (FFC) using modified multivariable Poisson regression analysis for prediction of DAWBA (Development and Wellbeing Assessment) interview outcomes 3 years later.

**Results:**

One unit’s increase in YSR was estimated to correspond to an increase in the relative risk of a probable DAWBA-based diagnosis by 3.3% [RR (95% CI) 1.03 (1.03–1.04), p < 0.001]. In gender-specific analysis, the findings applied, particularly to girls.

**Conclusions:**

Youth Self-Report (YSR) scores at pubertal age predicted the risk of a probable mental diagnosis at the onset of adulthood, particularly in girls. Further research is needed to explain the lower sensitivity of YSR among boys.

## Background

The prevalence of mental disorders is on the rise, and in several previous studies, girls have reported a higher prevalence than boys [1, 2], particularly when it comes to internalized problems. Depression and anxiety concurrently represent the most prevalent forms of internalized problems [3, 4]. In addition, in a rather recent study, girls report a steeper increase in the prevalence of anxiety and depression than boys [1]. The higher prevalence of problems found in other studies indicates that more girls than boys will rate a higher prevalence of problems at 15 years; therefore, more girls will also receive a diagnosis at 18 years of age [5].

According to a British national follow-up cohort study [6], adolescent mental health problems predict future psychopathology and mental distress. Some studies have also focused on mental health problems in adolescence and the association with subsequent mental disorders according to the ICD-10 classification for planning and evaluation of treatments in clinical settings. [7]. The Avon Longitudinal Study of Parents and Children (ALSPAC), from the UK, has focused on mental health problems among children of the 1990s and current health issues [8]. They found a significant association between self-reported total difficulties and later mental disorders, especially in girls. Other studies have also compared the predictive power of self-reported data and parental ratings to validate different assessment scores [9, 10]. It has been established that the YSR should be a valuable source for predicting mental disorders [3]. In their national epidemiological study among 11^th^-grade students, the results showed a prevalence of total mental problems of 19.0% among girls (CI 15.8–22.2) vs. 15.7% among boys (CI 12.0–19.4).

Externalizing problems are commonly reported by parents using the Child Behavior Check List (CBCL) and by teachers using Teacher Report Form (TRF) [11]. However, to ensure consideration of adolescents’ perspectives, self-report is a common way of detecting mental health problems nowadays. A European review [12] showed that self-rated health was a significant predictor of mental- and physical-health in the 19 countries included. Mental health problems were measured by inquiring about the frequency of feelings of sadness and depression as internalizing problems. They also found corresponding gender differences in 11 of the 19 countries with significantly greater validity in the ratings among middle-aged women compared to men [12]. Moreover, when using a Development and Wellbeing Assessment (DAWBA) interview both in the community and in the clinic, the self-rating part among adolescents showed an odds ratio of 8 compared to a corresponding odds ratio of 27 reported by the parents [13].

The health behaviour of school-aged children (HBSC) study observed a better validity in girls than boys in case of psychological complaints [14], which are comparable to internalizing symptoms. Moreover, a study using the same items as the HBSC study found more self-rated psychological difficulties in girls between 13 and 22 years than in boys [15]. Also, 52% of girls and 30% of boys 11–15 years of age reported self-rated multiple health complaints in the international report of HBSC [14]. Another study using self-reports based on the Strengths and Difficulties Questionnaire (SDQ) suggested that internalizing emotional problems were more common among adolescent girls. At the same time, boys reported a higher frequency of externalizing behavioral problems [16]. Also, when looking at total difficulties, girls demonstrated a 50% higher prevalence of high scores (9 vs. 6% among boys, p < 0.01), which might indicate that girls have more often internalizing symptoms and do, to a greater extent, identify them as such. In a study looking at feelings of loneliness, asking directly ‘do you feel lonely?’ created more considerable gender differences than asking other types of questions relating to loneliness [17]. A gender difference in adolescence could imply that data collection using self-reports needs more critical and detailed scrutiny from a gender perspective, assuming that externalizing problems may be under-reported by the adolescents themselves and over-reported by parents and teachers. Internalizing problems seem to be more sensitively reported by adolescent girls [2, 5]. The concept of masculinity [18], in which a boy or a girl enacts a general set of expectations connected to sex roles in the cultural context, may be necessary. The socialization into roles assumes that men and women learn a gendered perspective of what it entails to be a man or a woman. According to Connell [18], men aspire to a hegemonic ideal of toughness, power, authority, and competitiveness. These attitudes do not go hand in hand with identifying personal difficulties. Masculinity and femininity are products of socialization or social learning within the context, and new generations usually reproduce these patterns.

YSR (Youth self-rating scale) include both internalizing and externalizing symptoms as girls identify internalizing symptoms while boys might have difficulties identifying externalizing ones. In a study of (n = 7912) children aged 5–16, the likelihood of a DAWBA- based diagnosis based on parents’ ratings varied among girls from OR = 0.7–0.8 compared to boys [19]. One reason for this could be that parents more easily identify externalized problems [2, 5] which are more common in boys. If self-reports are more sensitive in identifying internalizing than externalizing symptoms, arguably, girls are expected to rate themselves as having more problems than boys will. Externalizing problems might not be as easily observed in self-reports [11, 20]. Proxy-raters, such as parents or teachers, have fewer difficulties identifying externalizing issues. However, this gender difference is not expected to be apparent in a clinical interview when information from different sources is concomitantly available. A DAWBA interview will identify boys, but their self-ratings will not be as predictive as we can assume girls’ self-ratings to be. Arguably, girls should more efficiently identify their own internalized issues, and their self-reports ought to be more accurate in predicting later probable DAWBA-diagnosis than boys’ self-reports.

There is a significant public health interest in the early detection of subthreshold mental health problems to prevent them from developing into full-scale mental disorders; however, the question remains if it is possible to detect them among boys than girls as reliably. Concomitantly, during the last decades, adolescents’ mental health problems and disorder assessment methods have been developed at a population level. Some assessment tools are aimed at the general or specific parts of the population, whereas others are primarily designed for clinical settings [7]. To get the complete picture, it is common in research to collect data from several informants, such as teachers, parents, and health service professionals; sometimes, only a single source is available. A previous study [21] showed that parental assessment with the CBCL significantly predicted mental disorders at 15 years of age [22]. YSR is a self-reported assessment scale that is a part of the Achenbach System of Empirically Based Assessment (ASEBA) [23], as is the CBCL. It is a widely used instrument both in clinical and community settings to assess adolescent emotional and behavioral problems [24]. However, self-reported YRS as a predictor of subsequent adolescents’ risk of a mental diagnosis determined by the DAWBA interview has not previously been explored, especially not from a gender perspective.

This study aimed to estimate whether self-reporting mental health problems using YSR predicts the risk of incident mental diagnosis and if the findings apply to both genders. The first hypothesis is that adolescents with a high prevalence of self-reported mental health problems at 15 years of age show an increased risk of a mental diagnosis determined by DAWBA at 18 years of age. The second hypothesis is that girls will have higher YSR scores due to internalizing problems while boys will have higher scores for externalizing problems. However, the gender difference will possibly not be as visible in DAWBA interviews.

## Materials and methods

### Data collection

Data from the ongoing population-based Finnish Family Competence (FFC) study was used for the study. FFC used a stratified randomized cluster sample from the Province of Turku and Pori in Southwestern Finland [25]. The study population originally consisted of families visiting a maternity health clinic when expecting their first baby in 1986. Earlier studies have described the sampling procedure in detail, e.g., [25]. In the chosen geographical area, 1287 babies were born and included in the FFC study sample. In total, 1125 children and their families participated at the child’s age of 12 years (87%). At the age of 15 years, 838 (74%) adolescents responded to the YSR questionnaire. At the age of 18 years, 599 (53%) adolescents participated in a DAWBA interview. In all, 504 participants responded to YSR and participated in the DAWBA interview. Most adolescents lived in families with their biological parents, and only 9% lived in single-parent families. Parents gave informed consent at the beginning of the FFC Study, and adolescents consented at 18 years. The Joint Ethics Committee of the University of Turku and the Turku University Central Hospital approved the Finnish Family Competence Study design.

### Instruments

#### Youth self report

The Youth Self report (YSR) score is a tool for identifying mental health problems. The YSR measures psychosocial adaptation, which focuses on experienced skills and perceived problems for clinical screening and epidemiological research [23]. The score is part of the Achenbach System of Empirically Based Assessment (ASEBA)—a widely used instrument package of different scores with specific parts for parents, teachers, caregivers, and adolescents to rate. YSR is a self-reported tool concerning behavioral and emotional problems for individuals aged between 11 and 18 years, which allows the measurement of internalized and externalized problems and the total problem score. The questionnaire provides scores for the following eight syndromes: withdrawn, somatic complaints, anxious/depressed (these three comprise the scale of internalizing symptoms). Delinquent behavior, aggressive behavior (these comprise the externalizing scale); social problems, thought problems, and attention problems [23] are neither internalized nor externalized problems. However, the total score comprises all eight scales, i.e., all problems measured by YSR, including those not considered internalizing nor externalizing. A total of 102 questions such as: ‘I feel that no one loves me’, ‘I’m too fearful or anxious’, and ‘I like to try new things’ were included in the study. Responses from 0 = not true to 2 = very true were used based on the preceding 6 months of assessments. Missing values on more than 20 questions omitted that individual from the analysis. The YSR score is a validated scale used in numerous previous studies showing alpha values of α = 0.94 for Total problems, α = 0.86 for Internalized, and α = 0.86 for Externalized problems [5].

#### Development and well-being assessment scale

The development and well-being assessment (DAWBA) scale indicates single or multiple mental probable diagnoses. The DAWBA interview at the age of 18 was used to establish the risk of psychiatric diagnoses [13]. The DAWBA is an epidemiological, frequently empirically used research tool developed to generate probable comparable diagnoses in line with ICD-10 and DSV-IV [26]. The DAWBA applies the previous 4-week timeframe for all the disorders except for anxiety disorder (i.e., past 6 months). The DAWBA interview comprises a set of questionnaires focusing on emotional (internalizing), behavioral, and hyperactivity (externalizing) symptoms. Based on the responses, particularly for this purpose, trained psychiatric nurses used a computer-based evaluation program to determine a probable mental diagnosis or not. The DAWBA-based probable diagnosis comprised obsessive–compulsive disorder (OCD), generalized anxiety disorder (GAD), depression disorders, conduct disorders, social anxiety, agoraphobia, panic disorders, autism spectrum disorders, post-traumatic stress disorder (PTSD), and eating disorders, among others (www.dawba.info). The Finnish version was back-translated into English [18], and during the process, Goodman, the creator of the DAWBA scale, was consulted.

#### Background variables

Background variables based on the parental history were obtained during the first visit to the health service clinic. The variables included were: child’s gender (female vs. male), mother’s and father’s age at child’s birth, parents’ basic education (≤ 9 years vs. > 9 years), parents’ additional vocational education (≤ vocational school vs. college/university), and parents’ socioeconomic status (SES) measured according to professional status (blue-collar vs. higher).

### Statistical methods

The study used the Modified Poisson regression with univariate and multivariable analyses for binary data [27]. The dichotomous variable, ‘any DAWBA-based diagnosis’ vs. ‘no diagnosis’, ‘was set as the outcome variable to assess adolescents’ mental diagnosis at 18 years. Background variables were included as dichotomous covariates. YSR total sum was used as the principal continuous explanatory variable. By using single predictor models, the covariates’ associations with DAWBA were checked to refine YSR’s predictive ability. Some of the background variables found in earlier research [3, 28] were included in the multivariable models if p-values < 0.1 were found, which constituted an inclusion criterion. In this step, the model also included pairwise interactions of covariates with YSR to find whether the association between YSR and DAWBA would change concerning the covariates. Boys and girls were analyzed together as no gender interactions were found. None of the interactions reached the required significance level (p ≥ 0.14 for all); hence, the final multivariable models only consisted of main effects. The association between DAWBA and YSR remained after adjusting for the covariates mentioned above. The backward selection was used from the final multivariable model to exclude all non-significant p-values (p < 0.05). A dropout analysis including all covariates of the analyses between participants with and without DAWBA was carried out. The analysis showed that declining a DAWBA interview was more common among children of mothers with lower education and boys. The results are reported as risk ratios (RR) with 95% confidence intervals (CI). The SAS System for Windows, release 9.4 (SAS Institute, Cary, NC, USA), was used for statistical computations.

## Results

Most adolescents (n = 450 of n = 504, 89%) did not demonstrate any mental disorder. The total sample indicated a small overrepresentation of girls (n = 289 of n = 504, 57%). However, the overrepresentation in the probable diagnosis group (44 girls vs. 10 boys) is much larger than in the sample. Girls are more than three times more likely to receive a diagnosis at the age of 18 years compared to boys, 15.2 vs. 4.7%. This observation justified more detailed scrutiny of the results from a gender perspective despite the non-significant gender interactions. The most common disorders were depression 31%, social anxiety 19%, panic disorders 19%, and eating disorders 17%. The four participants with OCD were all girls; none of them had any other diagnoses. Furthermore, none of the boys had agoraphobia, GAD, or an eating disorder. More than 37% of the participants had more than one diagnosis. Table 1 presents the distribution of diagnoses according to gender.

**Table 1:** The profile of the DAWBA-based diagnoses at 18 years of age

	Frequencies (n)	Frequencies (%)	Girls (%)	Boys (%)
Depression	17	31	82	18
Social anxiety	10	19	80	20
Panic disorders	10	19	80	20
Eating disorders	9	17	100	0
Conduct disorders	6	11	50	50
PTSD	6	11	67	33
Agoraphobia	5	9	100	0
OCD	4	7	100	0
GAD	4	7	100	0

^a^The total number of diagnoses is higher than the number of adolescents with diagnoses since 34 persons were labeled with one diagnosis, 9 with two diagnoses, and 11 with more than three diagnoses

**Fig. 1:**
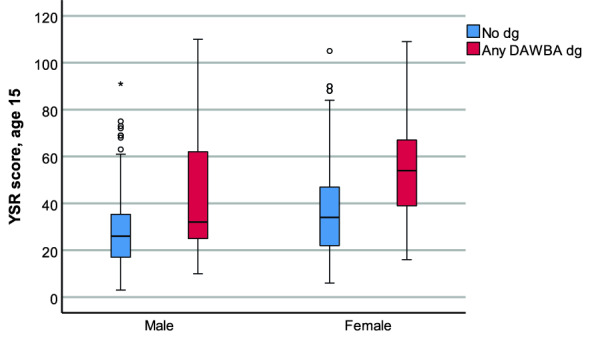
The distribution of median YSR scores concerning gender (male, female) and DAWBA-based diagnosis (no diagnosis, any DAWBA diagnosis)

Self-reported mental health problems, expressed as a higher YSR score at 15 years of age, proved to be the main risk factor for mental health 3 years later. The YSR-scores varied from 3 to 105 for those not receiving a probable mental diagnosis and between 10 and 110 for those receiving a probable diagnosis. The difference in median YSR scores between the group with a DAWBA-based diagnosis and the group without any diagnosis was 14 (diagnosis: median = 45 IQR = 29.0) vs. (no diagnosis: median = 31 IQR = 24.0). Those who were subsequently diagnosed reported more mental health problems at the age of 15. A closer examination of the graphical presentation of the results (Fig. 1) justifies closer consideration of boys with a further analysis regarding gender and its association with internalized and externalized problems.

The score of internalizing symptoms for girls varied between 3 and 42 (median = 18.0; IQR = 14.7), boys varied between 2 and 26 (median = 9.0; IQR = 12.0) in the DAWBA-based diagnosis group. For externalizing symptoms, the corresponding figures for girls were between 4 and 28 (median = 14.5; IQR = 9.5) and for boys between 2 and 42 (median = 13; IQR = 19.0), respectively. Girls in this group reported both more often internalizing and externalizing symptoms; however, the difference was more considerable regarding internalizing symptoms. DAWBA identified much fewer boys (n = 10) than girls (n = 44), whether internalizing and externalizing difficulties.

Regarding confounders, mothers of adolescents receiving a probable DAWBA-based diagnosis were younger than those not receiving one (no diagnosis median age = 26.5; IQR = 4.7 vs. diagnosis median age = 26.1; IQR = 5.9). Regarding fathers’ age, the difference was not as clear (no diagnosis median age = 28.9; IQR = 5.0 vs. diagnosis median age = 28.1; IQR = 5.6). Adolescents with a DAWBA-based diagnosis were more likely to have parents with lower education; for example, more blue-collar working fathers were in the diagnosis group than in the non-diagnosis group (12.4 vs. 6.1%). Background information is given in Table 2, and the distribution of YSR scores and gender are graphically illustrated in Fig. 1.

**Table 2:** Descriptive statistics on the two groups, created from ratings according to the Development and Well-being Assessment (DAWBA) Scale of 18-year-old adolescents and gender inclusive parental data at child’s birth, and Youth Self Report (YSR), at the age 15 years (n = 504)

	No diagnosis according to DAWBA n = 450	Mental health diagnosis according to DAWBA n = 54
YSR	Median (IQR) Mean (SD)	Median (IQR) Mean (SD)
YSR score at the age of 15 years	31.0 (24.0) 32.9 (18.1)	45.0 (29.0) 51.4 (23.0)
**Internalizing symptoms**	8.0 (9.0) 9.5 (6.8)	15.3 (15) 16.0 (9.5)
Girls	10.0 (9.0) 11.6 (7.5)	18.0 (14.7) 17.4 (9.5)
Boys	6.0 (6.0) 7.0 (4.8)	9.0 (12.0) 10.1 (7.5)
**Externalizing symptoms**	10.0 (9.0) 10.3 (6.6)	14.5 (10.0) 15.3 (8.0)
Girls	10.0 (8.0) 10.5 (6.3)	14.5 (9.5) 14.9 (6.7)
Boys	9.0 (9.0) 10.0 (6.9)	13.0 (19.0) 17.1 (12.5)

Gender	n (%)	n (%)
Girls (n = 289)	245 (84.8)	44 (15.2)
Boys (n = 215)	205 (95.4)	10 (4.7)

Parental data at child’s birth	Median (IQR) Mean (SD)	Median (IQR) Mean (SD)
Mother’s age (total n = 492)	26.5 (4.7) 27.0 (3.7)	26.1 (5.9) 25.9 (4.2)
Father’s age (total n = 464)	28.9 (5.0) 29.4 (4.2)	28.1 (5.6) 28.3 (4.8)

	n (%)	n (%)
Mother’s basic education		
> 9 years (n = 268)	241 (89.9)	27 (10.1)
< 9 years (n = 236)	209 (88.6)	27 (11.4)
Father’s basic education		
> 9 years (n = 159)	144 (90.6)	15 (9.4)
< 9 years (n = 345)	306 (88.7)	39 (11.3)
Mother’s vocational training		
College/university (n = 226)	205 (90.7)	21 (9.3)
< College/university (n = 278)	245 (88.1)	33 (11.9)
Father’s vocational training		
College/university (n = 157)	145 (92.4)	12 (7.6)
< College/university (n = 347)	305 (87.9)	42 (12.1)
Mother’s SES		
White-collar (n = 56)	52 (92.9)	4 (7.1)
< White-collar (n = 448)	398 (88.8)	50 (11.2)
Father’s SES		
White-collar (n = 132)	124 (93.9)	8 (6.1)
< White-collar (n = 372)	326 (87.6)	46 (12.4)

Results for the univariate and multivariable analyses are presented in Tables 3 and 4. The subscales for internalizing and externalizing problems were included concomitantly as explanatory variables in the final multivariable analyses. In the multivariable analysis, an increase of one-unit in self-reported mental problems at 15 years was related to an increased relative risk of a mental diagnosis at 18 years of 3.3%. Girls showed an increased relative risk of mental diagnoses at 18 years compared to boys (RR = 2.26), indicating a more than two-fold risk increase. YSR divided into internalizing symptoms showed an RR of 4% (CI 1.01–1.07), while externalizing symptoms showed an RR of 6% (CI 1.02–1.09). Gender in the model lowers the RR of internalizing more than externalizing scores because participants with high internalizing scores and subsequent probable DAWBA-based diagnoses were mainly female. While the parents’ low age was significant and the father’s SES was not quite significant in univariate analysis, these aspects were insignificant in the multivariable analysis indicating independent predictive power of YSR. YSR and female gender increased the likelihood of subsequent incidences of mental disorders. The median for no DAWBA-based diagnosis of girls is in line with the median of the boys who did receive a DAWBA-based diagnosis. As each incremental value of the YSR increased the risk for a later probable diagnosis, higher values of self-rated mental health problems at 15 years of age should be seen as a risk for later diagnosis.

**Table 3:** Risk ratios (RR) with 95% Confidence intervals (CI) for a Development and Well-being Assessment (DAWBA) based diagnosis of mental disorders among adolescents at 18 years of age regarding total scores of Youth Self Report (YRS) and covariates

	Univariate			Multivariable		
	RR	95% CI	p	RR	95% CI	p
YSR at age 15, 1-unit increase	1.03	1.02–1.04	< 0.001	1.03	1.02–1.04	< 0.001
Sex, girls vs. boys	3.27	1.69–6.36	< 0.001	2.26	1.18–4.33	< 0.014
Father’s age, 1-year decrease	1.06	0.98–1.15	0.152			
Mother’s age, 1-year decrease	1.08	0.99–1.18	0.070			
Father’s SES max blue-collar vs. higher	2.04	0.99–4.21	0.054			

A modified univariate and multivariable Poisson regression analysis

**Table 4:** Risk ratios (RR) with 95% Confidence intervals (CI) for a Development and Well-being Assessment (DAWBA) based diagnosis of mental disorder among adolescents at 18 years of age according to externalized and internalized sub-scale scores of Youth Self Report (YSR) and covariates

	Univariate			Multivariable		
	RR	95% CI	p	RR	95% CI	p
YSR Internalizing symptoms at age 15, 1-unit increase	1.08 7.6%	1.05–1.1	< 0.001	1.04	1.01–1.07	0.007
YSR Externalizing symptoms at age 15, 1-unit increase	1.08 7.7%	1.05–1.10	< 0.001	1.06	1.02–1.09	0.002
Sex, female vs. male				2.47	1.26–4.86	0.008

A modified univariate and multivariable Poisson regression analysis

## Discussion

The follow-up cohort study showed that an increase by one unit of the YSR score increased the overall relative risk of a DAWBA-based diagnosis by 3.3% 3 years later, given that the studied association is a linear one. The result confirmed both the first and the second hypothesis that self-reported mental health problems at 15 years of age could predict the risk of a probable mental diagnosis in 3 years of follow-up, specifically for girls. As one incremental point indicates one additional area with problematic issues or more pronounced problematic issues within one area. We may speculate that higher values of the YSR likely indicate multiple areas with problems or one area with more profound problems. The insensitivity of the YSR scores to detect subsequent mental diagnoses of boys could not be attributed to the fact that the number of observations as diagnoses generally was much lower among boys than the girls (Fig. 1).

As the sample of the study is population-based, our results are generalizable to teenage populations, and YSR can accordingly be used to identify typical youth for risk for future diagnosis. The precedent YSR scores showed a broader range in the diagnosis group. To the best of the researchers’ knowledge, no previous study has explored mental health problems and subsequent mental health in an epidemiological community setting using concomitantly the clinically validated assessment instruments YSR and DAWBA.

The DAWBA scale has been validated for predicting probable mental disorders according to ICD-10, e.g., [7]. DAWBA has shown strong associations with both ICD-10 and DSM-IV, but DAWBA diagnosed more comorbid disorders than the latter, especially in clinical settings [26]. The previous studies show that DAWBA can be a more readily applicable measure even in epidemiological and clinical settings [13, 29]. DAWBA was initially created as a screening tool for adolescents for psychopathology to support the planning of services [13]. Aebi et al. [9] compared the contribution of youth- and parent information using both DAWBA and the SDQ to identify mental disorders or problems in adolescents. They found that adolescents’ self-reports alone are reasonable substitutes for screening purposes in cases when the information from multiple sources like parents, teachers, and youth or healthcare services [9] is not available. Our study indicates that self-reporting, at least on a group level, shows that identifying mental health problems can be conducted based on single informants, at least in the case of girls. This might be central, especially if obtaining information from several informants is not possible.

Research, e.g., [9, 10], states that responses from multiple sources are preferable to clarify the current status of the adolescents’ mental status. Such sources include self-reporting, parental ratings, and teachers’ or other caregivers’ reports. Recently, we found that parental assessment, measured by CBCL at offspring’s age of 15 years, was independently and significantly predictive of an increased risk of 3% of the adolescent receiving mental diagnoses at 18 years of age [21]. The present study shows that self-report alone was, somewhat surprisingly, not more accurate but rather a comparable and similar predictor to parental assessment. The increased risk was of the same magnitude compared to the previous study [21] in which the informant was one of the parents, generally the mother.

Our study inferred that girls diagnosed with mental disorders more often than boys demonstrate a higher risk even when considering other factors. Girls reported externalized problems more than three-fold and internalized problems more than two-fold in the DAWBA-based diagnosis group compared to girls without a diagnosis. These results align with previous studies that found that girls have more emotional problems than boys [28]. Among 3446 Austrian adolescents, 16.5% reported emotional and behavioral problems using YSR, and clinically relevant internalized problems were reported more often (18%) than externalized problems (7%). Girls reported more total problems, internalized problems, and problems in all domains except somatic complaints, social, and thought problems. Externalized problems seemed to rise with increasing age for girls and even more for boys in the 11th grade [3]. A Finnish cohort study [30] that followed adolescents during two phases showed that the low socioeconomic status of the parents contributed more strongly to internalizing and externalizing behavior problems (measured by YSR) in female adolescents than in boys. Their focus was on investigations concerning parental socioeconomic status, adolescents’ screen time, and physical activities through externalizing and internalizing characteristics.

In recent decades, mental health problems in childhood or adolescence have been rated using various scales worldwide [31], such as DAWBA or the diagnosis system ICD-10. The YSR scale has predominantly identified adolescents’ psychiatric problems in clinical and community settings and evaluated medical treatment [32]. A longitudinal study of a community-based sample of adolescents 11-, 14-, and 17-years of age showed that parental educational level and single parenthood predicted emotional problems for both genders [28]. A comparative study on clinical and community settings found that adolescent girls reported higher scores on internalizing and externalizing disorders in clinical settings than girls originating in community settings [33]; therefore, lower YSR scores are expected in a community sample.

The gender difference found, especially regarding boys, needs to consider gender roles. Adolescents grow up in a society, and the prevailing norms shape their attitudes [18]. Finnish men are not exempted from being shaped by hegemonic masculinity, and this might carry the risk of poor mental health in boys [34] and impede men from seeking professional help for their mental health problems [35]. Arguably the mental illness discourse and masculinity are incompatible in that a masculine man cannot also have mental health problems. Both boys and girls act as the expectations of society and earlier generations. During the ongoing criminal investigation, a study concerning incarcerated male adolescents examined YSR scales’ ability to predict psychiatric disorders [36] and found that YSR was not useful in that context. Our results indicate lower usefulness for boys as well. Self-reported mental health problems by YSR are influenced by society, culture, and own interpretations of friends’ thoughts about what is expected of an individual. Also, it has to be considered that individuals can perceive the same behavior differently [10]. However, Rescorla et al. [5] found in their study comparing adolescents’ YSR scores in 24 countries that girls reported significantly higher scores on total problems and internalized problems, and boys reported higher scores on externalizing problems. They did not find significant gender differences in attention problems. Similar results were inferred by [2] in a later study. Boys may have more difficulties recognizing their mental health problems and tend to act out their mental health problems resulting in problematic consequences as girls again report more internalized problems [37]. Despite these studies, the final explanation for why girls report higher YSR scores is still unknown. The difference concerns particularly total problems and internalized problems scores raising the question of whether the social construction of masculinity and the expectation of gender [38, 39] is still, to some extent, neglected when screening for mental health problems among adolescents?

According to the present study, both the DAWBA interview and the YSR instrument are more sensitive in the case of girls than boys. Moreover, girls report their feelings and mental issues more actively than boys. Previous studies show that boys tend to underreport their feelings and use fewer health care services than girls [39]. This under-reporting might result from social constructionism of gender in society [40], particularly evident in the Nordic societies. The process may start in childhood, and it can be speculated that some tendencies that are already present at 15 years of age are more manifested at the age of 18. Parents, teachers, and health care staff possibly do not note boys’ internalizing symptoms as sensitively as externalizing symptoms. Masculinity enacts even healthcare personnel, teachers, and other adults in the adolescents’ surroundings [18]. In case boys underreport their mental health problems, one consequence could be that we miss relatively more boys at risk for subsequently developing a mental diagnosis in screenings than girls. Similar gender differences might exist in other well-known psychological or neuropsychological assessment instruments, and further studies are still needed.

Parents’ low socioeconomic status is a risk factor for the offspring’s mental health [3]. For instance, in the present study, the fathers’ SES had a borderline significant relation in the univariate analysis but not in the multivariable analysis. This relation indicates that adolescents’ self-reported mental health problems could predict mental disorders even after controlling for parental socioeconomic status. In previous studies, biological and sociocultural factors and parental mental health were interpreted to contribute to the risk. Low socioeconomic status and living in single-parent families were associated with higher problem scores regarding mental health problems [3]. This relation aligns with a national cohort study, which found that low parental socioeconomic position during childhood was associated with later mental diagnosis in adolescents and adulthood [41]. However, a low parental SES was less sensitive to later mental problems than adolescents’ self-reports.

A major limitation, common in all longitudinal studies, is the decline in retention rates. The attrition might have been strengthened in the present study because new informed consent was needed from adolescents before the DAWBA interviews. Responses from nearly 40% of the original sample with adolescent data from YSR and DAWBA can still be considered satisfactory as considerable attrition is inevitable in long-term prospective follow-up studies. A dropout analysis showed that omitting DAWBA was more common among boys and children of mothers with lower education. This may have mitigated the result but does not justify the interpretation that the study’s main finding, i.e., the predictive power of YSR towards future DAWBA, would be biased. The same applies to the gender differences detected in the study. In the present study, it was not possible to examine associations between YSR and a specific DAWBA-based diagnosis because of the low number of specific DAWBA-based diagnoses. Also, DAWBA generates fewer diagnoses than other standardized diagnostic interviews [26, 29]. Finally, the quantification of the increase of the risk of a probable mental diagnosis by 3.3% for each YSR score presupposes a linear association. However, because receiving a mental diagnosis is a rare phenomenon, the linearity could not be verified, and the quantification should be understood as an approximation.

## Conclusions

Youth Self-Report (YSR) scores on mental health problems at pubertal age contribute to risk prediction of a probable mental diagnosis at onset of adulthood, and this applies particularly to internalized problems in girls. Further research is needed to explain the lower sensitivity of YSR among boys, especially in the case of externalized problems.

## Data Availability

The data the study is based on contains personal and intimate information that, according to initial agreements with the participants from the start of the follow-up, cannot be transferred to third parties or outside the borders of Finland. Theoretically, some subsets of data could on reasonable request be delivered but unfortunately, in most cases, it probably would be evaluated as not possible along with this. The datasets used and analyzed during the current study are available from the corresponding author on reasonable request.

## Abbreviations

ALSPAC: Avon longitudinal study of parents and children
ASEBA: Achenbach system of empirically based assessment
CBCL: Child behavior check list
DAWBA: Development and well-being assessment
DISC: Diagnostic interview schedule for children
DSV-IV: Diagnostic and statistical manual of mental disorders, 4th edition
FFC: Finnish family competence
GAD: Generalized anxiety disorder
HBSC: Health behaviour of school-aged children
ICD-10: International classification of diseases, 10th revision
OCD: Obsessive-compulsive disorder
PTSD: Post-traumatic stress disorder
SDQ: Strengths and Difficulties Questionnaire
TRF: Teacher Report Form
WHO: World Health Organization
YSR: Youth Self Report
